# Jaborandi in the Treatment of Asthma

**Published:** 1881-02

**Authors:** H. J. Thomas

**Affiliations:** Greenwood, Wis.


### ARTICLE IX.

GREENWOOD, WIS., Dec. 28, 1880.

EDITORS JOURNAL AND EXAMINER,

*Dear Sirs:*—I have noticed, lately, two articles in your journal on asthma, and as I have had some experience with that stubborn complaint, I wish to offer a suggestion in its treatment, which has been of service to me in a good many cases, and has indeed never failed being of some benefit to my patients.

It is the use of jaborandi. I saw some time since an article in which it was recommended for asthma. At that time I was using it (as I also was giving all the "new remedies" a trial) in my general practice. I happened to prescribe it in one case (for other symptoms), where the patient had suffered for years with asthma. She told me as she was becoming convalescent, that she believed I was curing her of her asthma. Knowing how freely it excites mucous secretions, also having noticed its relaxing effect on involuntary muscular fiber (for I have used it in colic and even for tenesmus of dysentery), I concluded it must be the jaborandi she had taken, that had produced the good effect in her case. I continued its use, making almost a complete cure. Since then I have treated about twenty patients with *this alone*, that number being all the asthmatic patients within about fifty miles of me; and not one has it failed to help. I give 4-drop doses, fluid extract, middle of forenoon, middle of afternoon, at about 7 o'clock p. m., and 8 or 10 drops at bedtime. Should these doses produce a very free flow of saliva, then I give a little less, but *follow it up faithfully* four times a day, even repeating the evening dose in the night, should a paroxysm come on.

Give this a trial, fellow practitioners, and let us hear, through the JOURNAL, your success with it. (Of course, in anæmic cases I gave constitutional treatment, chalybeates, etc., in addition.) I will respond promptly to any personal letter from regular physicians on this subject, for I am more than positive of the good results it has given me.

H. J. THOMAS, M.D.

